# Cervical Spine Hyperextension and Altered Posturo-Respiratory Coupling in Patients With Obstructive Sleep Apnea Syndrome

**DOI:** 10.3389/fmed.2020.00030

**Published:** 2020-02-07

**Authors:** Louis Clavel, Ségolène Rémy-Neris, Wafa Skalli, Philippe Rouch, Yoann Lespert, Thomas Similowski, Baptiste Sandoz, Valérie Attali

**Affiliations:** ^1^Sorbonne Université, INSERM, UMRS1158 Neurophysiologie Respiratoire Expérimentale et Clinique, Paris, France; ^2^Arts et Métiers, Institut de Biomécanique Humaine Georges Charpak (IBHGC), Paris, France; ^3^AP-HP, Groupe Hospitalier Universitaire APHP-Sorbonne Université, site Pitié-Salpêtrière, Service de Pneumologie, Médecine Intensive et Réanimation (Département R3S), Paris, France; ^4^AP-HP, Groupe Hospitalier Universitaire APHP-Sorbonne Université, site Pitié-Salpêtrière, Service des Pathologies du Sommeil (Département R3S), Paris, France

**Keywords:** obstructive sleep apnea syndrome, biplanar X-ray, personalized 3D models of the spine, posturo-respiratory coupling, respiratory emergence, cervical spine hyperextension, head forward, hyperkyphosis

## Abstract

Obstructive sleep apnea syndrome (OSAS) is associated with postural dysfunction characterized by abnormal spinal curvature and disturbance of balance and walking, whose pathophysiology is poorly understood. We hypothesized that it may be the result of a pathological interaction between postural and ventilatory functions. Twelve patients with OSAS (4 women, age 53 years [51–63] (median [quartiles]), apnea hypopnea index 31/h [24–41]) were compared with 12 healthy matched controls. Low dose biplanar X-rays (EOS® system) were acquired and personalized three-dimensional models of the spine and pelvis were reconstructed. We also estimated posturo-respiratory coupling by measurement of respiratory emergence, obtaining synchronized center of pressure data from a stabilometric platform and ventilation data recorded by an optico-electronic system of movement analysis. Compared with controls, OSAS patients, had cervical hyperextension with anterior projection of the head (angle OD-C7 12° [8; 14] vs. 5° [4; 8]; *p* = 0.002), and thoracic hyperkyphosis (angle T1–T12 65° [51; 71] vs. 49° [42; 59]; *p* = 0.039). Along the mediolateral axis: (1) center of pressure displacement was greater in OSAS patients, whose balance was poorer (19.2 mm [14.2; 31.5] vs. 8.5 [1.4; 17.8]; *p* = 0.008); (2) respiratory emergence was greater in OSAS patients, who showed increased postural disturbance of respiratory origin (19.2% [9.9; 24.0] vs. 8.1% [6.4; 10.4]; *p* = 0.028). These results are evidence for the centrally-mediated and primarily respiratory origin of the postural dysfunction in OSAS. It is characterized by an hyperextension of the cervical spine with a compensatory hyperkyphosis, and an alteration in posturo-respiratory coupling, apparently secondary to upper airway instability.

## Introduction

Obstructive sleep apnea syndrome (OSAS) is characterized by instability of upper airways, which leads to their intermittent obstruction during sleep (1, 2). The frequency of obstructive respiratory events, as well as the intermittent hypoxia and fragmented sleep that results (1, 2), are strongly correlated with excessive daytime sleepiness, an increased risk of accidents, and the development of cardiovascular, metabolic and neurocognitive co-morbidity. Ventilation with continuous positive airway pressure (CPAP) remains the mainstay of treatment in moderate to severe OSAS, preventing obstructive respiratory events (3), reducing daytime sleepiness (4), the risk of accidents (5) and increased cardiovascular risk (6–8).

Recently, a specific postural dysfunction has been reported to be a feature of OSAS. Reported series describe abnormal spinal curvature, with cervical hyperextension and anterior projection of the head (9–11), disturbances of balance (12, 13), and walking (12, 14, 15), and an increased risk of falls (16). These difficulties with balance and walking improve significantly after treatment with CPAP is instituted, suggesting that they are specifically related to OSAS (12, 14). It would seem that there is also a causal link between OSAS and anterior projection of the head. Patients with known OSAS generally acquire this in childhood, and it worsens during growth if OSAS remains untreated (9), and is notably more marked if OSAS is severe (11). Despite this, no study has correlated the anatomical postural anomalies and disturbances of balance, nor is the pathophysiology of this postural dysfunction well-understood. In particular, its association with intermittent hypoxia and altered sleep quality, the principal factors that lead to co-morbidity, remains purely theoretical. Indeed, though changes in stabilometric parameters have been correlated with sleep deprivation (17) or to profound hypoxia (18), which is not typically observed in studies of OSAS patients (12, 13). Additionally, the intermittent hypoxia and altered sleep quality do not explain the hyperextension of the cervical spine and the forward projection of the head (9–11). The cervical hyperextension most often found in adolescents and young adults who present with a reduction in the posterior pharyngeal space (10), are evidence of a relationship between the angle of the head, the mechanical properties of the upper airways, and ventilation. Should cervical flexion induce an increase in upper airway resistance (19), hyperextension increases the pharyngeal diameter (20), flow rate in the upper airway, and its stability when awake (21), and thus facilitates ventilation in the healthy subject (21), and in patients in whom oral ventilation predominates (22). In OSAS patients the upper airways resist the flow of air (23), and cervical hyperextension with anterior projection of the head seems to facilitate ventilation (20), representing a postural adaptation to pathology of respiratory origin. In this context, we hypothesized in this study that postural dysfunction of OSAS is the result of a pathological interaction between postural function and ventilatory function that causes both problems of static posture and disturbances of balance.

Both these functions are in fact physiologically linked. Anatomically, both rely on the integrity of the same musculoskeletal structures: the spine, thoracic cage, and trunk musculature. It has been shown that spinal curvatures are severely constrained by extreme variations in pulmonary volume (24). Additionally, normal ventilation cyclically perturbs balance. Spinal curvature varies during the ventilatory cycle because of change in the orientation of the costo-vertebral joints (25). This “postural disturbance” caused by ventilation is manifested in stabilometric recordings as minimal oscillations of the center of pressure whose ventilatory origin is suggested by their disappearance on breath-holding/apnea (26), and an increase in voluntary (27), or hypercapnic (28) ventilation. The end result is that the cyclical constraint imposed on the thoracic spine by normal breathing continually changes the head to pelvis vertical axis, requiring the body to compensate (29) at the neck and pelvis (24) to maintain normal postural balance. Each ventilatory cycle triggers spinal and pelvic contractions to compensate for this postural disturbance (30–32) and is evidence for the existence of a posturo-respiratory coupling that is in play across all joints from head to foot when standing. This coupling is centrally controlled (31, 33) and may be affected by cortical lesions such as those following a cerebro-vascular accident. In patients with such lesions, disturbances of balance are correlated with an increase in postural dysfunction of ventilatory origin (34).

In the context of the pathological posturo-respiratory interaction which is central to the hypothesis of our study, we would expect to detect alterations of spinal alignment which may interfere with the maintenance of vertical balance (29, 35), an increase in postural dysfunction of ventilatory origin, and/or reduced effectiveness in the mechanisms that counter this disturbance (31). In order to explore this, we studied posturo-respiratory coupling in a group of OSAS patients and matched controls (27), using stabilometric recordings and non-invasive monitoring of ventilation using a motion capture system. Spinal curvature, head posture, and pelvic orientation in the erect position were also captured, modeling postural alignment via the acquisition of biplanar radiography and individualized 3D skeletal reconstructions (36).

## Materials and Methods

### Inclusion Criteria

#### Patients

Patients with moderate to severe OSAS, with a diagnosis in the 6 months prior to study entry based on symptoms and polysomnography, without known postural dysfunction or other respiratory pathology, were included. Patients treated with CPAP or mandibular advancement device (MAD) had to stop their treatment at least 1 week before the study procedures.

#### Control Group

Healthy non-snoring subjects with a low probability of OSAS (scoring 0 on the Berlin questionnaire) (37), were included in the control group.

Participants of both group had to have a normal respiratory function as well as neurological and musculoskeletal examination. No participant should have had a history of falls in the 12 months prior to study entry.

Ethical approval for the study in both OSAS patients and healthy subjects was obtained from the relevant bodies (the Comité de Protection des Personnes (CPP) Ouest V on 28 June 2017 and the CPP Ile de France VI on 18 February 2015). The study was registered in the ISRCTN register (Registration numbers ISRCTN70932171 for the OSAS patients, ISRCTN56129394 for the healthy subjects). All participants were informed of the nature of the study and gave written consent to participate.

### Study of Postural Alignment Using 3D Biplanar Imaging

**Figure 1:**
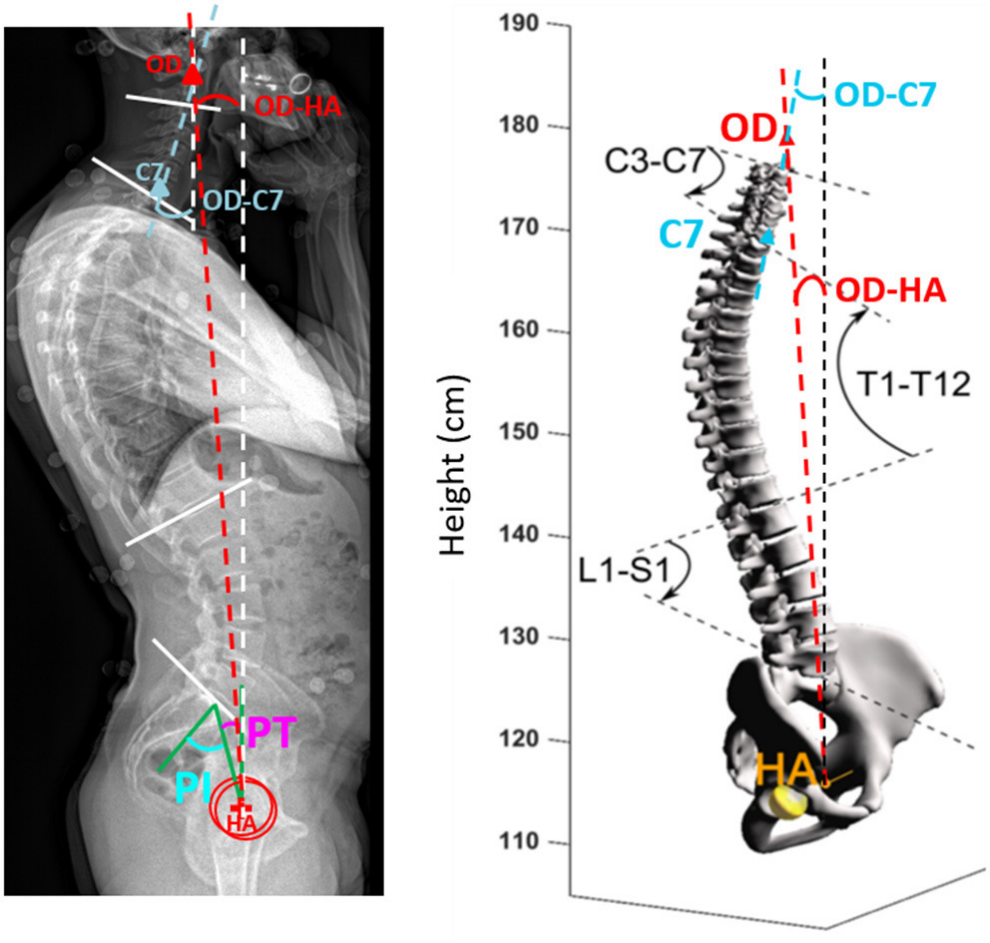
Analysis of postural alignment using 3D biplanar imaging. PT pelvic tilt, PI pelvic incidence, OD superior tip of the odontoid process of C2, OD-HA the angle between the vertical plane and the line through OD and the midpoint of the line connecting the center of the two femoral heads, OD-C7 the angle between the vertical plane and the line through OD and C7. C3–C7, 3D cervical curvature; T1–T12, 3D thoracic curvature; L1–S1, 3D sacral curvature.

The EOS® system (EOS® Imaging, France) was used to simultaneously acquire bi-planar (sagittal and coronal) images of the entire skeleton (36, 38). Participants were placed in a reference position with their hands on their cheeks while breathing normally. Individual 3D models were constructed from the biplanar imaging, including the points OD, defined as the uppermost point of the odontoid peg of the second cervical vertebrae (C2), the spine of the third cervical vertebrae (C3) to the sacrum (S1), and the pelvis, using established methods (39, 40). The following values were then calculated: (1) 3D spinal curvatures: cervical, between C3 and C7 (C3–C7 angle), thoracic, between T1 and T12 (T1–T12 angle), lumbar, between L1 and S1 (L1–S1 angle), expressed in degrees; (2) pelvic variables (pelvic incidence and pelvic tilt); (3) the angle between the vertical and a line drawn from OD to the mid-point of a line intersecting the femoral heads (OD-HA); (4) the angle between the vertical and a line drawn from OD and the uppermost point of C7 (OD-C7) (see Figure 1).

### Study of Posturo-Respiratory Coupling

#### Acquisition and Measured Parameters

The simultaneous acquisition of ventilatory and stabilometric profiles were performed by using an optico-electronic system of movement analysis (Vicon with Nexus 2.5, Oxford, UK), which was synchronized with a force platform (BP 4051040-2K, AMTI, Watertown, USA). Sixty-five reflective markers were positioned on each participant (41 on the thorax, four on the head, seven on each leg, and three on each foot). The ventilatory profile, the antero-posterior movement of the cervical spine, and the antero-posterior rotation of the pelvis, knees, and ankles were captured using 12 cameras positioned around the participant, with an acquisition frequency of 100 Hz. The participants stood on a force platform that simultaneously measured the trajectory of horizontal displacement of the pressure center (PC) projected along the antero-posterior (AP, sagittal) and medio-lateral (ML, coronal) axes, defined for each subject using six markers placed on the feet, with an acquisition frequency of 100 Hz.

#### Experimental Protocol

Data was acquired with the subject in a relaxed upright position, with both feet positioned on the force platform the width of the pelvis apart, with the arms at the sides. Participants were asked to maintain horizontal gaze by looking at a point located at eye height on the wall opposite. After a period of familiarization with this environment, recordings were obtained while breathing naturally in sequences lasting 1 min for two visual conditions: eyes open (EO) and eyes closed (EC) and two occlusal conditions: occlusion (OC; with the mandible positioned by teeth contact), and rest position (RP; with the mandible positioned by the muscles without tooth contact). The different conditions were presented in random order.

### Parameters Analyzed

Respiratory frequency (RF), inspiratory time (Ti), expiratory time (Te), and the cinematic angles of the cervical spine, pelvis, knees, and ankles were calculated from the movement analysis. The amplitude of displacement of the pressure center along the AP (AP Range) and ML (ML Range) axes was calculated. Using the time-synchronized data from the movement analysis (ventilatory signals and the variation in cinematic angles of the cervical spine, pelvis, knees, and ankles) and the stabilometric analysis, posturo-respiratory coupling was then calculated using the respiratory emergence (REm) method described by Hamaoui et al. (27). The REm is defined as the ratio of the power frequency extracted at a 0.08 Hz frequency band centered on the average ventilatory frequency of the pressure center displacement signal. This variable, which may have values between 0 and 100, estimates the contribution of the ventilatory component to the cinematically-observed movements, with higher values indicating a greater contribution of the ventilatory component. It is calculated from the displacement of the pressure center along the AP and ML axes, and by anatomical segment (cervical spine/pelvis/knees/ankles).

### Statistical Analysis

As none of the parameters are normally distributed, results are expressed as medians with inter-quartile intervals. The data from both groups were compared using the Mann-Whitney test for discontinuous data and by Fisher's exact test for qualitative variables. Postural alignment was also compared to corridors of normality previously published (29).

## Results

### Participants

Twelve patients with OSAS and twelve healthy subjects were included. Participants in both groups were similar in age, gender, and body mass index (BMI). Two of the OSAS patients had received no treatment, and ten had been able to stop their treatment with CPAP or MAD for 10 [8–11] days. Patient characteristics and the results of the respiratory function testing in both groups are presented in Table 1.

**Table 1:** Baseline characteristics and pulmonary function tests.

	**OSAS Patients *n* = 12**	**Controls *n* = 12**	***p***
**BASELINE CHARACTERISTICS**			
Gender M/F	8/4	8/4	1
Age (years)	53 [51; 63]	50 [47; 60]	0.45
Height (m)	1.73 [1.65; 1.79]	1.74 [1.66; 1.76]	0.95
Weight (kg)	80 [73; 94]	76 [69; 79]	0.11
BMI (kg/m^2^)	27.8 [24.9; 30.5]	25.5 [23.7; 26.0]	0.07
Epworth	10 [8; 14]	6 [3; 8]	0.03
AHI (/h)	30 [21; 41]	ND	–
AI (/h)	13 [8; 19]	ND	–
Oxygen desaturation index (/h)	21 [12; 24]	ND	–
Time with oxygen saturation <90% (%)	8 [1; 17]	ND	–
PULMONARY FUNCTION TESTS (HELIUM DILUTION TEST)^*^			
VC L (% predicted)	5.0 [3.4; 5.9] (120 [114; 126])	5.0 [4.1; 5.5] (122 [116; 129])	0.79
IC (% predicted)	3.8 [2.8; 4.2] (136 [119; 161])	3.4 [2.8; 4.2] (133 [124; 139])	0.98
ERV (% predicted)	0.9 [0.7; 1.5] (81 [50; 98])	1.4 [1.2; 1.9] (116 [77; 136])	0.10
FRC (% predicted)	2.9 [2.1; 3.6] (89 [80; 99])	3.0 [3.0; 3.8] (101 [89; 111])	0.37
RV (% predicted)	2.0 [1.7; 2.2] (97 [79; 103])	1.8 [1.5; 2.0] (89 [78; 94])	0.45
TLC (% predicted)	6.3 [5.0; 7.4] (106 [99; 111])	7.0 [5.7; 7.4] (105 [103; 110])	0.60

*Data are expressed as medians with inter-quartile*.

*BMI, body mass index; VC, Vital Capacity; IC, Inspiratory Capacity; ERV, Expiratory Reserve Volume; FRC, Functional Residual Capacity; RV, Residual Volume; TLC, Total Lung Capacity; AHI, apnea hypopnea index; AI, apnea index; ND, not done*.

* *Pulmonary function tests performed in all controls, and in 11/12 patients, helium dilution test performed in all controls, and 10/12 patients*.

### Postural Alignment

**Figure 2:**
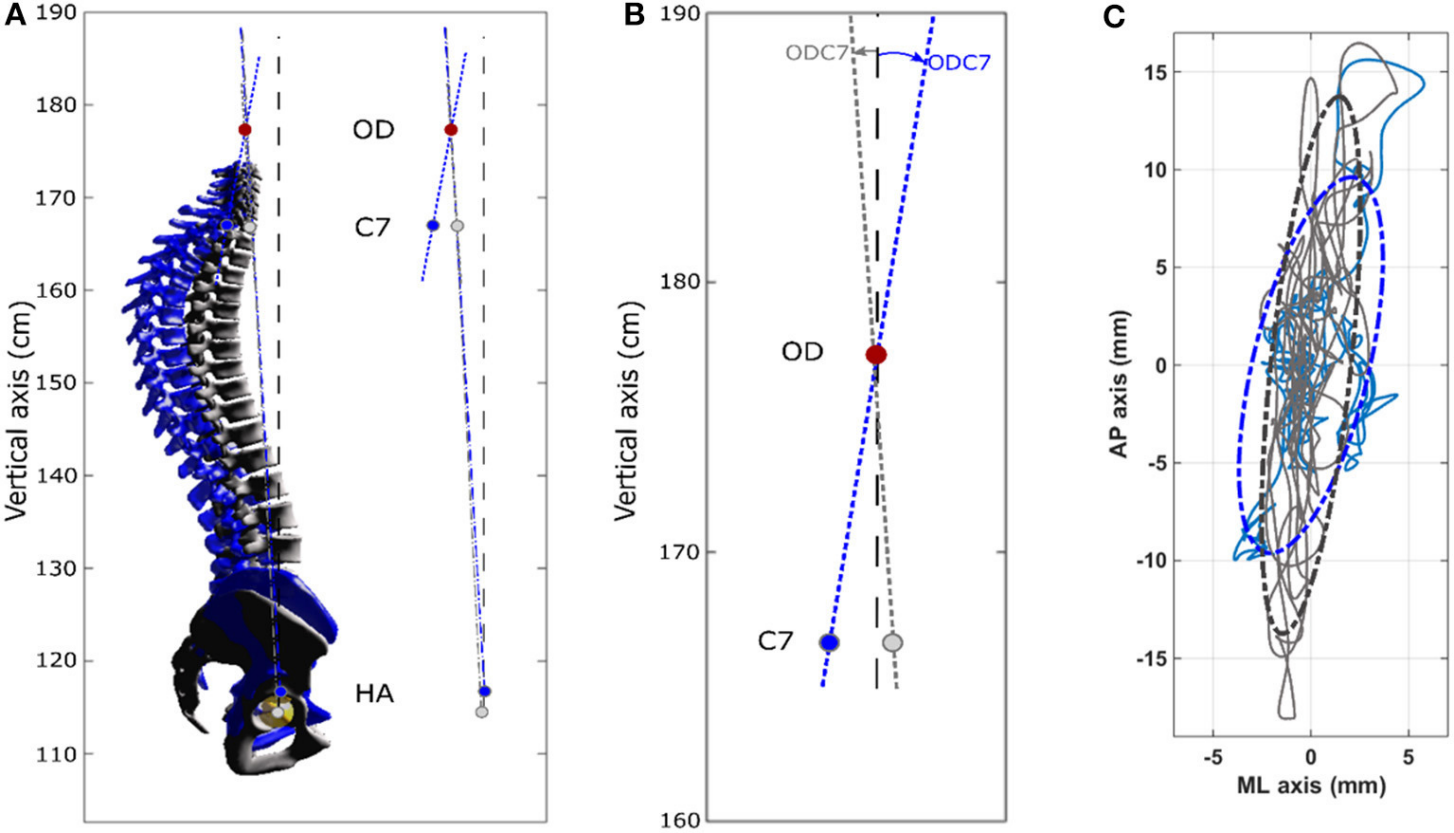
Spinal alignment obtained by 3D reconstruction (EOS) and stabilometric profile obtained from OSAS patient n°7 (female, 53 years, 1 m59, 67 kg) control subject n°9 (female 48 years, 1 m62, 55 kg). In the OSAS patient compared to the controls: thoracic hyperkyphosis and cervical hyperextension is seen; center of pressure displacements are lower in the antero-posterieur axis and greater in the medio-lateral axis. **(A)** Left: 3D spinal reconstructions. OSAS patient, blue (3D spinal angles: C3–C7 = −27.2°, T1–T12 = 77.4°, L1–L5 = −44.8°) Control subject, gray (3D spinal angles: C3–C7 = −1°, T1–T12 = 38.5°, L1–L5 = −46.3°). Right: sagittal projection of OD-HA and OD-C7 angles. The OD-HA (3D) angle is 3.1° in the OSAS patient and 2.9° in the control subject. **(B)** Enlarged view of the sagittal projection of the OD-C7 angle, which is 8.3° in the OSAS patient and 3.4° in the control subject. **(C)** Center of pressure displacements. OSAS patient (blue) and control subject (gray). The 95% confidence interval is represented by the dotted ellipses.

The overall alignment with respect to the vertical (OD-HA angle) was similar in both groups: the head was almost exactly vertically aligned above the pelvis in both groups (see Table 2). However, the spinal curvatures differed significantly between patients and controls. In OSAS patients, we observed a greater OD-C7 angle, which represents evidence of upper cervical hyperextension (Table 2). We also observed more marked thoracic kyphosis in OSAS patients (Table 2), with six patients having values greater out of the corridor of normality (superior to 63°) (29), compared with only one control (*p* = 0.069). Finally eight OSAS patients had lower cervical kyphosis (C3–C7) compared with only one control (*p* = 0.01). Figure 2 shows an example of postural alignment in a patient, and in matched control subject.

**Table 2:** Spinal and pelvic parameters derived from 3D reconstruction parameters (EOS parameters).

	**OSAS**	**Controls**	***p***
**EOS PARAMETERS**			
C3–C7 (°)	8 [−13; 11]	−7 [−10; −4]	0.24
T1–T12 (°)	65 [51; 71]	49 [42; 59]	0.039
L1–S1 (°)	−60 [−63; −52]	−58 [−62; −47]	0.41
OD–HA (°)	4 [1; 4]	3 [3; 5]	0.59
OD–C7 (°)	12 [8; 14]	5 [4; 8]	0.002
Pelvic incidence (°)	53 [43; 57]	55 [47; 62]	0.80
Pelvic tilt (°)	15 [12; 24]	13 [11; 16]	0.41

*Data are expressed as medians with inter-quartile*.

*OD-HA, angle between the vertical and the line that connects the most superior point of the odontoid peg of the center of the bi-coxofemoral axis; OD-C7, angle between the vertical and the line that connects the most superior point of the odontoid peg and the seventh cervical vertebrae; SVA, Sagittal Vertical Axis; C3–C7, cervical curvature between the third cervical vertebrae to the seventh; T1–T12, thoracic curvature between the first thoracic vertebrae and the twelfth; L1S1, lumbar curvature between the first lumbar vertebrae and sacrum*.

### Stabilometric and Ventilatory Parameters

The stabilometric profile of the patient and control groups differed significantly in all conditions (Table 3). In the EO/RP condition, we observed a significant difference in the amplitude of displacement of the pressure center between the groups. The OSAS patients' displacements were lower in the AP range, and greater in the ML range (example in a patient, see Figure 2). This difference was also observed in the three other experimental conditions, though not significantly so in the EC/RP condition. Both groups had similar ventilatory parameters (Ti, Te, RF) in all experimental conditions (Table 3) and there was no significant intra-group variation in the various experimental conditions.

**Table 3:** Posturo-respiratory interaction as a function of visual and occlusion conditions.

**Condition**	**E0/RP**			**EC/RP**			**EO/OC**			**EC/OC**		
	**OSAS**	**Controls**	***p***	**OSAS**	**Controls**	***p***	**OSAS**	**Controls**	***p***	**OSAS**	**Controls**	***p***
RF (/min)	13.2 [10.7; 14.6]	14.0 [12.7; 17.3]	0.143	15.5 [11.4; 16.3]	13.3 [12.0; 16.5]	0.143	14.6 [13.3; 15.5]	15.3 [13.0; 17.2]	0.319	11.6 [10.9; 13.4]	14.5 [12.1; 17.0]	0.089
Ti (s)	2.5 [2.1; 2.9]	2.0 [1.8; 2.2]	0.060	2.0 [1.9; 2.8]	2.2 [2.0; 2.4]	0.713	2.1 [2.0; 2.4]	2.0 [1.9; 2.2]	0.078	2.7 [2.2; 3.0]	2.1 [1.8; 2.4]	0.052
Te (s)	2.4 [2.0; 3.1]	2.5 [2.0; 2.8]	0.713	2.1 [1.9; 2.9]	2.4 [2.0; 2.8]	0.887	2.2 [2.1; 2.4]	2.2 [1.7; 2.7]	0.670	2.8 [2.4; 3.0]	2.2 [1.9; 2.6]	0.089
Range AP (mm)	1.5 [0.4; 3.5]	7.1 [1.8: 19.0]	0.033	2.4 [1.1; 5.3]	9.6 [1.2; 23.5]	0.101	1.9 [0.8; 3.1]	6.6 [1.6; 13.9]	0.052	1.4 [0.5; 2.9]	7.6 [2.5; 27.9]	0.010
Range ML (mm)	19.2 [14.2; 31.5]	8.5 [1.4; 17.8]	0.008	26.4 [20.7; 38.6]	7.7 [2.7; 19.4]	0.005	17.7 [13.8; 25.0]	8.9 [1.7; 20.1]	0.114	22.7 [14.8; 31.8]	9.2 [2.4; 16.5]	0.021
Rem AP (%)	13.0 [9.5; 19.8]	12.8 [6.3; 21.2]	0.799	11.1 [6.0; 17.4]	11.8 [7.4; 16.1]	0.799	8.0 [5.6; 16.7]	8.0 [5.4; 11.3]	0.843	10.3 [8.1; 14.2]	16.2 [9.1; 23.0]	0.160
Rem ML (%)	19.2 [9.9; 24.0]	8.1 [6.4; 10.4]	0.028	21.3 [10.8; 34.2]	10.0 [6.9; 13.0]	0.039	17.7 [11.7; 27.4]	7.2 [2.8; 12.4]	0.017	20.4 [14.4; 34.2]	8.1 [6.5; 11.5]	0.005

*Data are expressed as medians with inter-quartile intervals. EO, Eyes Open; EC, Eyes Closed; OC, Occlusion (mandible positioned by the contact of the teeth); RP, Rest Position (mandible positioned by the muscles without occlusion); RF, respiratory frequency; Ti, inspiratory time; Te, expiratory time; Range AP, amplitude of center of pressure displacement on anteroposterior axis; Range ML, amplitude of center of pressure displacement on mediolateral axis; Rem AP, respiratory emergency on anteroposterior axis; Rem ML, respiratory emergence on mediolateral axis*.

### Respiratory Emergence

**Figure 3:**
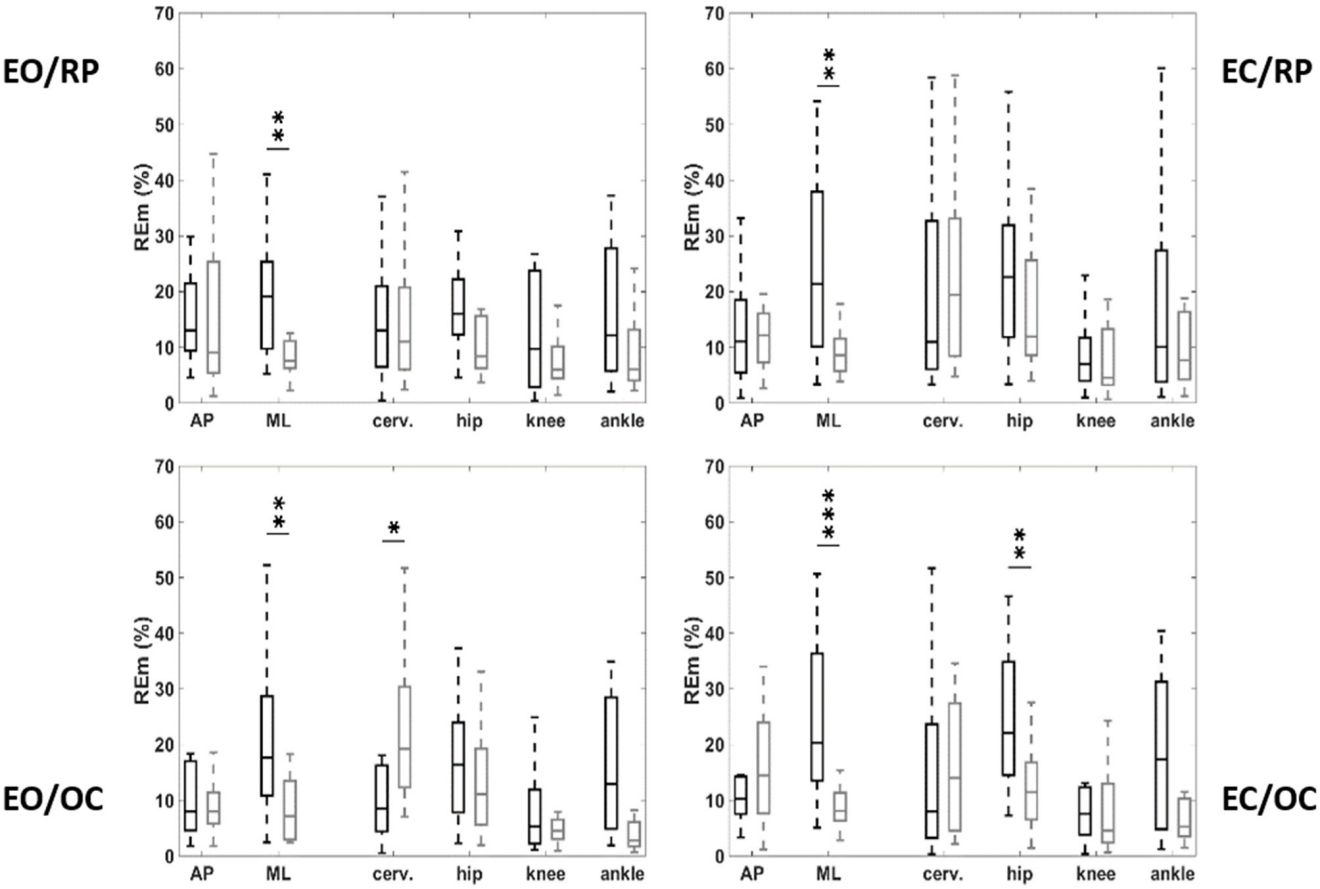
Respiratory emergence on antero-posterior (AP) and mediolateral (ML) deplacement of the center of pressure, and variation of kinematic angle at the level of the cervical spine, pelvis, knees, and angles, as function of vision and occlusion. OSAS patients (black), controls (gray). EO, eyes open; EC, eyes closed; OC, mandible positioned by occlusion of the teeth; RP, mandible positioned by the muscles without occlusion. *p < 0.05; **p < 0.01; ***p < 0.001.

We observed a significant increase in respiratory emergence, and in consequence, posturo-respiratory coupling, represented by greater displacement of the pressure center due to respiratory movements, along the medio-lateral axis in the EO/RP conditions in OSAS patients compared with controls (19.2% [9.9; 24.0] vs. 8.1% [6.4; 10.4]; *p* = 0.028), an increase that was also reproduced in the three other conditions (Table 3). Analyzing by segment, we noted significant differences between patients and controls only in the conditions when the mandible was in a position of occlusion (EO/OC and EC/OC). In OSAS patients respiratory emergence was less at cervical level in the EO/OC condition (8.6% [4.7; 15.7] vs. 18.1% [13.8; 28.2]; *p* = 0.015) and greater at hip level in the EC/OC condition (11.7% [6.7; 21.9] vs. 22.1% [16.0; 34.7]; *p* = 0.045) (Figure 3).

## Discussion

This study provides evidence that OSAS patients have specific postural dysfunction, associating disturbances of balance and postural anomalies, characterized by hyperextension of the upper cervical spine, anterior projection of the head, and thoracic hyperkyphosis. The increases in postural displacement due to respiratory movements are evidence for the centrally-mediated and primarily respiratory origin of this postural dysfunction.

### Upper Airways and Spinal Alignment in OSAS

There is a close physiological relationship between spinal curvature and the stability of the upper airways because of the direct anatomical support that the cervical spine provides to the oropharynx. Cervical flexion increases the risk that the upper airway will collapse, while cervical extension increases its stability (19). This relationship is illustrated in certain upper cervical pathologies such as the osteochondromas, osteophyte formation, multi-focal lesions in rheumatoid arthritis or posterior subluxation of C1. Should these cause cervical flexion, and thereby exert pressure on the posterior wall of the pharynx, they may be directly responsible for obstructive respiratory events during sleep (41). The reductions observed in the apnea/hypopnea index, or even complete resolution of OSAS, that follows surgical restoration of normal cervical alignment, attests to a causal relationship (42). Equally, prior surgical procedures that achieved anterior access to the cervical spine constitute an anatomical risk factor for OSAS (43), because of the reduction in diameter of the upper airway which they are apt to engender. In our OSAS patients we did not observe cervical pathology causing flexion deformities that may potentially cause upper airway instability. Rather, we saw upper cervical hyperextension reflected in an increased OD-C7 angle. This adaptive posture has been previously described in OSAS (9). It is adaptive to ventilatory need to the degree that it facilitates awake ventilation, by improving the mechanical properties of the upper airways in the healthy subject (21), in mouth-breathers (22) and possibly in OSAS (20). Thus, the cervical hyperextension probably allows the body to compensate for the increased respiratory load when awake, induced by an alteration in the mechanical properties of the upper airways as OSAS develops (23, 44). Spontaneous correction of cervical hyperextension when awake OSAS patients are fitted with a mandibular advancement device to widen and stabilize the upper airways constitutes independent evidence for this hypothesis (45).

However, the upper cervical hyperextension and anterior projection of the head are potentially problematic for the maintenance of balance (35). We have already established that, in OSAS patients, the head-pelvis alignment remains close to the vertical, with a normal OD-HA angle (36), however the compensation to maintain this alignment seems different to that observe in the elderly (29). Indeed, we did not observed in our patients any pelvic compensation (29). Conversely we observed a compensatory thoracic hyperkyphosis (24, 29). This compensation probably limits the disturbance of balance secondary to pathological spinal alignment (35), but may increase the risk of falls and others hyperkyphosis-related comorbidities (46). Moreover, the change in head rotation may theoretically cause disturbances of balance by perturbing central processing of visual and vestibular afferents (47). In our study the compensation for postural disturbance of ventilatory origin during the mandibular occlusion condition observed in OSAS patients is evidence for the recruitment of mandibular proprioceptive afferents dedicated to the control of balance, supporting this hypothesis. The role of mandibular afferents is normally negligible in healthy subjects (48), and becomes increasingly important in dysfunctions of visual or vestibular control (49). The change in head position may equally also interfere with central perception of vertical posture (50) or alter cortico-cortical connections and the anticipatory adaptations of postural control by the pre-motor cortex (47), which in both conditions may lead to disturbances of balance.

### Changes in Posturo-Respiratory Coupling in OSAS

We have shown that there are significant differences between the stabilometric profiles of OSAS patients and controls. These results are coherent with previously published studies which have provided evidence that OSAS patients have poorer balance compared with a control population (12, 13). Additionally, we have now shown that this increase in CP displacement amplitude specifically concerns the ML axis. The control of CP displacement along this axis is primarily cortical (51), and the stabilometric data observed in the OSAS patients in our study support the presence of a postural disturbance of central origin. In this context, hypoxic cerebral lesions causing disturbances of balance have been described in OSAS (52, 53) and cannot be excluded in our patients, even in the absence of any known past neurovascular problems. However, we have equally also shown that the increase in CP amplitude along the ML axis is associated with an increase in respiratory emergence along this axis, suggesting a specifically respiratory origin for this postural disturbance. Our results are evidence for a change in central interaction between respiratory and postural functions (i.e., posturo-respiratory coupling) (27, 31, 33), and therefore suggest a possible link with the waking respiratory cortical adaptation previously reported in OSAS (54). The pre-inspiratory potentials observed in the motor cortex and the supplementary motor area during resting ventilation provide evidence for a pathological adaptive increase in awake cortical respiratory drive (55), which compensates for the intrinsic respiratory load linked to increased upper airway resistance, be this physiological in dorsal decubitus in the healthy subject (56), or linked to intrinsic loads in the abnormal upper airways of the OSAS patient avoiding obstructive events when awake (54). It has also been shown that respiratory cortical adaptation is associated with increased consumption of cognitive and attentional resources (57). In OSAS this may alter postural balance by competing for cortical resources, as control of balance and responses to inspiratory load are modulated in the same cortical areas (47). Such a mechanism has also been demonstrated when healthy subjects have had their inspiratory load increased experimentally (58). The exacerbation of gait abnormalities observed when OSAS patients are asked to perform a double task (e.g., performing a Stroop test whilst walking on a treadmill) (12, 14), demonstrates the close relationship between inspiratory load, locomotion, and cognition (58) and provides independent support for this hypothesis.

### Methodological Conditions and Study Limitations

The limited number of participants and the inclusion of non-obese patients mean that our results cannot be confidently generalized to all patients with OSAS. We recognize that the absence of polysomnographic data in the control group means that we cannot formally exclude the absence of OSAS in controls. However, our control subjects were completely asymptomatic. None snored or complained of somnolence, and their Berlin scores all equaled zero, a result whose negative predictive value has been calculated to be 72% using data from the Hypnolaus cohort (1, 59). However, we can reasonably consider this group to be composed of healthy subjects. We also recognize that we did not measure awake increase in upper airway resistance and collapsibility in our patients, and the calculation between the alteration of the mechanical properties of the upper airways and postural dysfunction remains theoretical. Correction of posturo-respiratory coupling and cervical hyperextension with a daytime mandibular advancement device (60) could contribute further evidence to complement this study.

## Conclusion

This study provides evidence for abnormal spinal alignment and disturbances of balance in OSAS patients, and calls for these to be sought in clinical practice in order to mitigate their consequences. The determination of posturo-respiratory coupling allows early screening for postural dysfunction and to refine understanding of its OSAS-related character. Finally, the potential correlation between the specific postural dysfunction of OSAS, the changes in the mechanical properties of the upper airways, and respiratory cortical adaptation to waking and cognitive problems, mean that correcting mechanical anomalies of the upper airways should be considered.

## Data Availability Statement

All datasets generated for this study are included in the article/supplementary material.

## Ethics Statement

The studies involving human participants were reviewed and approved by—the Comité de Protection des Personnes (CPP) Ouest V on 28 June 2017 for the OSAS patients. Number 17/018-2; ID RCB 2017-A00721-52 -the comité de protection des personnes CPP Ile de France VI on 18 February 2015 for healthy subjects. Number 90-06; ID RCB 2006-A000386-45. The patients/participants provided their written informed consent to participate in this study.

## Author Contributions

VA, LC, BS, and TS contributed substantially to the study design, data analysis and interpretation, and the writing of the manuscript. WS, PR, SR-N, and YL contributed substantially to the data analysis and interpretation, and the writing of the manuscript. All authors approved the final version of the manuscript and agreed to be accountable for all aspects of the work.

### Conflict of Interest

VA reports personal fees from ADEP Assistance, personal fees from Resmed, personal fees from Nyxoah, outside the submitted work. TS reports personal fees from AstraZeneca, France, personal fees from Boerhinger Ingelheim, France, personal fees from GSK, France, personal fees and non-financial support from Novartis, France, personal fees from Lungpacer Inc., Canada, personal fees from TEVA, France, personal fees from Chiesi, France, grants from Air Liquide Medical Systems, France, personal fees from ADEP Assistance, France, all outside the submitted work. The remaining authors declare that the research was conducted in the absence of any commercial or financial relationships that could be construed as a potential conflict of interest.
